# Implementing Subtle Clinical Indicators in Geriatric Assessments

**DOI:** 10.1177/30495334251330682

**Published:** 2025-03-31

**Authors:** Ali Ahmad, Halina Kusz, Mashael Hussain

**Affiliations:** 1 McMaster University, Hamilton, ON, Canada; 2 Brightshores Health System, Owen Sound, ON, Canada; 3 Michigan State University, East Lansing, MI, USA; 4 University of Calgary, Calgary, AB, Canada

**Keywords:** aging, communication, functional assessment, pain, psychosocial

## Abstract

As the medical landscape evolves, healthcare providers are increasingly faced with the challenge of balancing patient engagement with administrative demands. This shift underscores the critical need to prioritize meaningful interactions with patients, particularly within the context of geriatric care. This review examines the significance of subtle cues often overlooked in the geriatric population, including non-verbal communication, clothing, nails, and odor. These subtle indicators, when approached with sensitivity and keen observation, can reveal important information about the physical, psychological, and social well-being of older adults. We introduce the term “Subtle Clinical Indicators (SCI)” to encapsulate these observations, and recommend their integration into routine geriatric assessments. By attentively observing and documenting SCI, healthcare providers can better understand health conditions, personalize interventions, and adopt a more insightful approach to geriatric care.

## Introduction

Healthcare is evolving with technological advances and providers find their time diverted from meaningful patient interaction to ancillary work, administrative duties, billing and record keeping. It is important to return to mindful patient care and close observations. Medical education emphasizes history-taking and physical examination skills; however, it overlooks the importance of subtle observations which can offer valuable insights especially in the context of geriatric care (Keifenheim et al., 2015).

Traditional patient assessments focus on objective findings, leaving subtle observations overlooked, poorly documented, or even unaddressed. These cues can provide crucial insights into underlying health conditions, potentially preventing further decline (Fitzgerald & Tierney, 1982). This is especially important in older adults who may struggle to express concerns, particularly those with cognitive impairment (Murman, 2015).

Therefore, we propose introducing the term “Subtle Clinical Indicators (SCI)” into geriatric assessments. This will allow providers to be mindful of the findings, which may include non-verbal communication, eye contact, distress signs, attire, accessories, and odor. Of these, it is particularly important to be attentive to non-verbal cues which may include facial gestures, voice modulation, and use of interpersonal space. Providers should actively seek these cues and document their observations, enhancing the depth of patient assessment and plans.

## Non-verbal Communication

Nonverbal communication is especially important for clinicians interacting with older adults. Reading body language and understanding nonverbal cues can offer valuable insight. This is important as patients may face challenges in verbal communication due to cognitive decline (Hall et al., 2019). It is important to maintain a patient-centered approach, incorporating both verbal and nonverbal communication.

Clinicians should be attentive to both positive and negative nonverbal signs which can provide clues to medical diagnoses and treatment plans. It is therefore important to be mindful of facial expressions, voice changes, and use of interpersonal space. It is especially important with baby boomers, who depend more on body language than words. Perceiving these cues contribute to a more through assessment (Hall et al., 2019; Wanko Keutchafo et al., 2022). Non-verbal cues such as changes in posture, gait, and grooming habits can be significant indicators of depression among baby boomers.

When such observations are made, it is important to also document and follow up on them on future visits. It should also encourage an empathetic approach. For example, using an appropriate tone and pausing to allow patients time to respond enhances non-verbal communication (Pinedo-Torres et al., 2023).

## Poor Eye Contact

Providers should be attentive to a decrease in eye contact. This is particularly important if it is a change from previous interactions. Decreased eye contact may be due to vision impairment, which may prevent an individual from maintaining eye contact (Welp et al., 2016). Age-related vision loss, cataracts, glaucoma, and macular degeneration are all possible factors affecting eye contact (Quillen, 1999). Also, an underlying neurological cause, such as a history of stroke, or photosensitivity linked to migraine disorder, may lead to reduced eye contact (Digre & Brennan, 2012; Fruhmann Berger et al., 2006).

In specific instances, reduced eye contact may indicate cognitive impairment. For example, frontal lobe involvement may lead to apathy and reduced awareness of social cues. It may also be seen with advanced dementia (Lopis et al., 2019). Psychological factors, including social anxiety, major depressive disorder, grief, adjustment disorder, and social isolation, may also present with poor eye contact (Howell et al., 2016). In addition, social isolation can make it difficult for individuals to engage in social interactions.

While poor eye contact may be indicative of an underlying medical or psychological condition, personal, cultural, and religious factors can also influence an individual’s comfort level with eye contact. In certain instances, patients may intentionally avoid making eye contact with their clinicians as a form of respect particularly when there are differences in gender or social status (Juckett, 2005).

Nonverbal aspects of communication, such as eye contact, between clinicians and patients play a significant role in impacting medical outcomes, including patients’ understanding and adherence to treatment plans (Richard Street, 1988). In the clinical setting, it is advisable for clinicians to incorporate nonverbal cues through body language. It is important to address patients directly, using concise sentences and maintaining a natural tone of voice for effective interaction (Juckett, 2005).

## Signs of Distress

Prior to examining the patient, there may be early indicators of pain or distress. For instance, a patient might be dealing with lower back discomfort resulting from extended sitting in the waiting area. For individuals with limited mobility, the effort involved in attending the appointment may be considerable, possibly leading to signs of fatigue, such as closing their eyes (H. Singh et al., 2011).

Throughout the interview, the patient may express discomfort by way of grimacing, guarding, or vocalizations (Helmer et al., 2020). Since it is difficult to detect pain in patients with cognitive impairment, tools such as the PAIN-AD (Pain Assessment in Advanced Dementia), Abbey Pain Scale and CNPI (Checklist of Nonverbal Pain Indicators) have been developed (Kim et al., 2017). These instruments examine characteristics indicative of distress, including vocalization, grimacing, bracing, rubbing, restlessness, verbal complaints, labored breathing, facial expressions, tense body language, fidgeting, and distractibility (Carezzato et al., 2014; Corbett et al., 2014; Okimasa et al., 2016; Takai et al., 2010). While the scale does not need to be completed, providers should be familiar with what signs to look for.

Even in patients without cognitive impairment, clinicians should be vigilant for the manifestation of these expressions. It is crucial not to dismiss and rather to acknowledge that they are present, and explore further.

## Clothing

Clothing is also a subtle cue and can offer insights that beyond initial impressions. The way they dress may be reflective of their background and overall health. Stains and wear on their clothes can offer information about their past occupations. Accessories like ties and scarves become indicators of hobbies and health history.

Individuals may make certain clothing decisions to make it easier and more practical for them. For example, clinicians should pay attention to details like snaps and zippers. For instance, wearing clothes with zippers rather than buttons may make it easier for an individual to get ready. It is important for those with reduced digital dexterity caused by conditions like arthritis or motor diseases (Fitzgerald & Tierney, 1982). Also, the neatness of their appearance may reflect their overall well-being. In addition, color selection may offer insight. For example, unconventional color pairing may suggest color blindness (Fitzgerald & Tierney, 1982).

While some indicators may be evident upon the practitioner’s first glance, a dedicated effort to systematically observe the patient’s attire during the physical examination is crucial. Notably, loose or oversized clothing can signify significant health considerations, such as weight loss or alterations in body composition. Conversely, tight clothing may point to increased weight gain or fluid build-up, offering additional diagnostic clues (Fitzgerald & Tierney, 1982).

A particularly telling detail is the fit of a patient’s belt, which, when loose, may hint at underlying issues. New belt holes may be due to increased abdominal girth, possibly from ascites, or weight gain over time (Fitzgerald & Tierney, 1982). Also of note is that individual preferences, cultural influences, fashion choices, and religious practices may contribute to the chosen attire. Changes in weight that could be linked to malnutrition, unhealthy diets, shifts in appetite, or the presence of both acute and chronic illnesses. Additionally, loose clothing may signal muscle atrophy, physical deconditioning, or clinical frailty (Alibhai et al., 2005; Fitzgerald & Tierney, 1982).

Of notable concern is the potential impact of cognitive impairment, particularly in patients living independently. This may manifest in various ways: for example, wearing clothes an incorrect order such placing a t-shirt over a sweater, wearing incomplete attire (e.g., one sleeve only), wearing clothes backward, and layering excessively inconsistent with temperature. These instances highlight the different ways in which cognitive decline may impact the everyday task of dressing (Aleksandar Matic et al., 2010).

In cases of cerebral dominance, such as in patients with history of prior stroke, there may be increased wear or tear on the shirt cuff corresponding to the dominant hand side (Fitzgerald & Tierney, 1982).

In certain instances, loose clothing may serve as an indicator of dehydration, prompting a comprehensive examination for related signs such as dry mouth, parched lips, diminished skin turgor, and sluggish capillary refill (Morley & Kraenzle, 1994). To the contrary, tight clothing may suggest fluid overload, and findings such as jugular venous distension, dependent edema, and inspiratory crackles on lung exam may provide more insight (Claure-Del Granado & Mehta, 2016).

## Shoes

Observing a patient’s shoes may offer insight as well. It is important to look at the type of shoes, heel, and arch support. In some individuals, high heels may be the cause of their back pain. An individual who wears one slipper and one shoe or an open-toed shoe may be due to underlying conditions such as gout, trauma, arthritis, or bunions on the foot (Fitzgerald & Tierney, 1982). Sometimes individuals may prefer shoes without laces or untied laces for comfort especially if there is inflammation or edema of their foot.

Patients that present to the clinic with untied shoelaces may have underlying conditions making it difficult for them to tie their laces. Fore example, chronic lumbosacral pain, arthritis and neurological conditions such as Parkinson’s disease can make it a challenging task. Close observation such as the wear pattern on shoe soles may provide cues of gait and help distinguish subacute versus chronic hemiparesis. Also, observing patients socks which may have blood stain may point to trauma from peripheral neuropathy (Fitzgerald & Tierney, 1982).

## Jewellery

Jewellery reflects personal choice, however it may be reflective of an individual’s socioeconomic status and stylistic preferences. Subtle indicators, such as a ring that is either excessively tight or loose and enveloped in a bandage, may allude to significant fluctuations in weight, potentially attributed to fluid retention or loss. Also, the presence of accessories like pins, medallions, or cuff links can serve as visual narratives, providing glimpses into the wearer’s personal interests and professional pursuits (Fitzgerald & Tierney, 1982).

## Carry-Around Items

Sometimes, items that an individual is carrying in their hands can provide valuable clues to an individual’s lifestyle and preferences. For example, a lengthy novel may speak to both their interest and their educational level. An individual who carries a notebook or a daily planner may be reflective of their meticulous nature, however it can also be a sign of underlying amnestic changes. Some patients are into using technology, and have the latest phone, smart watch, wearable fitness technology (such as a fitness tracker to monitor steps). Recurrent use or mention of these during an appointment suggest individual is adept at using them, and may indicate reasonable cognitive functioning. Their dedication to fitness and lifestyle measures may also be inferred. However, in some cases an individual may struggle to use their phone, or rely on their care partner to provide information form their phone. This may be a hint at cognitive changes, which may be formally tested in another visit.

## Nail Changes

Despite its significance, close observation of nails is often overlooked. While an exhaustive exploration of nail pathologies is beyond the scope of this review, it should be considered part of SCI. Specifically, untrimmed or discolored nails may provide insight on an individual’s personal hygiene and social habits (Maddy & Tosti, 2018; Wollina et al., 2016).

Untrimmed nail may hint at functional decline, due to challenges in maintaining personal hygiene. Factors contributing to this may encompass physical limitations, arthritic changes, pain, intentional tremor, and diminishing mobility (Badley et al., 1984). Additionally, the lack of attention to nail care may be an indication of cognitive impairment in older adults (Shimokihara et al., 2022). Those facing cognitive challenges may either forget to address their nails, or in cases of advanced dementia, they might have lost the ability to do so.

Furthermore, the lack of nail maintenance can be reflective of personal neglect, particularly in patients experiencing depression and decreased motivation for personal care (Maloy, 2016; G. Singh et al., 2005). It may also be due to caregiver neglect, particularly in individuals who are highly dependent on others for daily activities (Yon et al., 2017). The consequences of untrimmed nails extend beyond esthetics, elevating the risk of injury and subsequent infections, underscoring the need for timely intervention.

Approaching these observations with sensitivity is important, and requires a comprehensive assessment of the patient’s overall health, living conditions, and support systems. Understanding the implications of untrimmed nails enables healthcare professionals to delve into potential health issues, facilitating a more holistic approach to patient care.

## Poor Body Odor

In older adults, the manifestation of poor body odor serves as a potential indicator of underlying health conditions, environmental factors, or challenges in personal care. The underlying causes warrant careful consideration in the clinical assessment (Hart, 1979).

A common contributor to poor body odor is inadequate personal hygiene practices, which may cause difficulties in activities such as bathing, grooming, or changing clothes. Physical limitations, notably impaired mobility, can make it difficult for individuals to shower independently, leading to a reliance on external assistance (De-Rosende-Celeiro et al., 2019). Patients may exhibit caution, fear of falls, which could result in a reluctance to bathe (Lachman et al., 1998). The fear of falling often leads to limiting one’s activities. One of the most pronounced instances of this fear is linked to the act of taking a bath in a tub (De-Rosende-Celeiro et al., 2019). In instances where daily assistance is lacking, the maintenance of personal hygiene may be compromised, underscoring the importance of support networks.

Advanced cognitive impairment represents another significant factor, where individuals may unfortunately lose the ability to care of themselves, further affecting their personal hygiene (Carpenter et al., 2006). Additionally, poor body odor may signify personal neglect, reflective of underlying depression, or caregiver neglect, in those individuals who are highly dependent on others (Mauk, 2011).

It is also important to recognize that poor body odor may be due to an underlying infection (Shirasu & Touhara, 2011). Fungal or bacterial skin infections, particularly in skin folds, should be considered. Furthermore, the possibility of an infected ulcer, especially in patients with limited mobility, necessitates investigation to rule out decubitus ulcers (Anders et al., 2010). Additionally, urinary or fecal incontinence, particularly in those struggling with personal hygiene management, may be a causative factor (Schnelle & Leung, 2004).

Nutritional factors, medication side effects, and underlying health conditions such as kidney and liver disease can also contribute to poor body odor. The distinct odors of substances like alcohol, tobacco, or cannabis should be considered, offering potential insights into the individual’s social habits (Shirasu & Touhara, 2011).

## Halitosis

Halitosis, or mouth odor, may reveal suboptimal oral hygiene practices, marked by insufficient brushing, flossing, and dental care (Li et al., 2023; Memon et al., 2023). This olfactory concern may manifest as a consequence of chronic gum disease, gingival inflammation, cavities, and, in certain cases, dental abscess. In the context of older patients utilizing dentures, halitosis can signify improper cleaning and maintenance, fostering food residue accumulation, and bacterial overgrowth (Memon et al., 2023).

Moreover, poor mouth odor may be attributed to dry mouth, a condition influenced by factors such as dehydration, reduced salivary flow, and medication side effects, particularly from anti-cholinergic and diuretic medications. Chronic health conditions, including Parkinson’s disease, neuromuscular disorders, and autoimmune conditions like Sjogren’s Syndrome, may contribute to dry mouth and, subsequently, halitosis (Barbe et al., 2017; Bollen & Beikler, 2012).

Beyond the physiological factors, halitosis may serve as an indicator of broader issues. It can be a consequence of personal neglect stemming from cognitive impairment, depression, or caregiver neglect. Additionally, nutritional deficiencies, substance use, including tobacco and alcohol, may be reflected in the characteristic odor of the breath (Mokeem, 2014; Strączek et al., 2023).

## Summary

This review underscores the significance of subtle clinical indicators in the geriatric population, elucidating their diagnostic potential for healthcare professionals. It is important for providers to be attentive to overlooked cues such as poor eye contact, clothing details, untrimmed nails, poor body odor, and halitosis. It may lead to important insights into the physical, psychological, and social well-being of older individuals. By observing these details, healthcare providers can enhance their ability to detect underlying health conditions, enabling tailored interventions and fostering a more holistic approach to geriatric care. This comprehensive analysis aims to demonstrate the diagnostic potential inherent in these unassuming yet crucial aspects of geriatric patient assessments, contributing to the advancement of geriatric healthcare.

**Table:** Subjective Clinical Indicators.

**Domain**	**Subjective Clinical Indicators (SCI)**	**Interpretation**	**Plan**
**Non-verbal Behavior**	Facial expressions, tone of voice, posture, gait, space	Depression, cognitive decline, anxiety	Empathic approach, mood screen, neuro assessment
**Eye Contact**	Reduced or avoidant gaze, change from baseline	Vision loss, dementia, withdrawal, cultural norms	Vision check, cognitive screening
**Signs of Distress**	Grimacing, guarding, vocalizing, fidgeting	Pain, discomfort, fatigue	Use PAIN-AD scale, explore underlying cause
**Clothing**	Disheveled, mislayering, tight/loose clothing	Cognitive decline, frailty, malnutrition	ADL and nutritional assessment
**Shoes & Socks**	Mismatched shoes, worn soles, untied laces	Neuropathy, Parkinson’s, pain, neglect	Gait and foot care assessment
**Jewellery**	Loose/tight rings, medallions, symbolic pieces	Weight change, fluid retention, personal history	Rapport building, Treat underlying cause
**Carried Items**	Books, planners, tech devices	Lifestyle clues, cognitive capacity	Assess independence, cognition
**Nails**	Long, discolored, neglected nails	Depression, dementia, poor mobility, neglect	ADL assessment, caregiver evaluation
**Body Odour**	Strong or musty odor	Infection, poor hygiene, cognitive or physical decline	Skin check, hygiene support
**Breath Odour**	Halitosis, dry mouth	Poor oral care, meds, systemic illness	Oral hygiene exam, hydration, med review
